# Supplementary material for: Decursin and Decursinol Angelate Suppress Adipogenesis through Activation of β-catenin Signaling Pathway in Human Visceral Adipose-Derived Stem Cells
Source: Nutrients. 2019 Dec 19;12(1):13. doi: 10.3390/nu12010013 (PMC7020042; doi:10.3390/nu12010013)
Supplement: Supplementary file 1 [file nutrients-12-00013-s001.pdf]

a

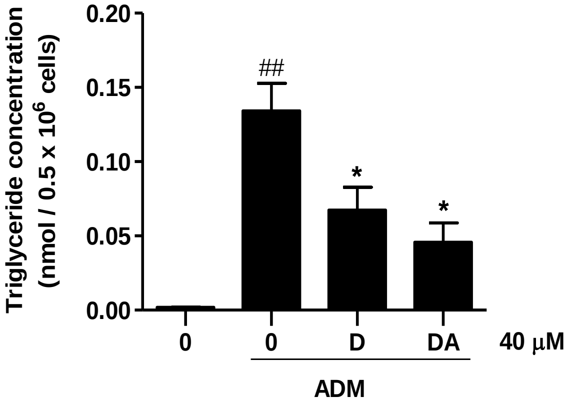

b

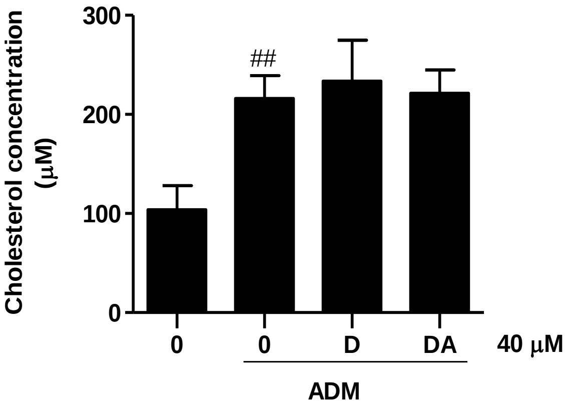

Figure S1. The effect of D and DA on the triglyceride and cholesterol deposition in ASCs. **(a)** The triglyceride concentration analyzed by triglyceride quantification assay on day 12 after ADM treatment. **(b)** The cholesterol concentration analyzed by cholesterol assay on day 12 after ADM treatment. Values are expressed as mean ± SD of three independent experiments. $^{\#\#}P < 0.01$ vs. undifferentiated group, $^{*}P < 0.05$ vs. ADM treated group. ADM: adipogenic differentiation medium;

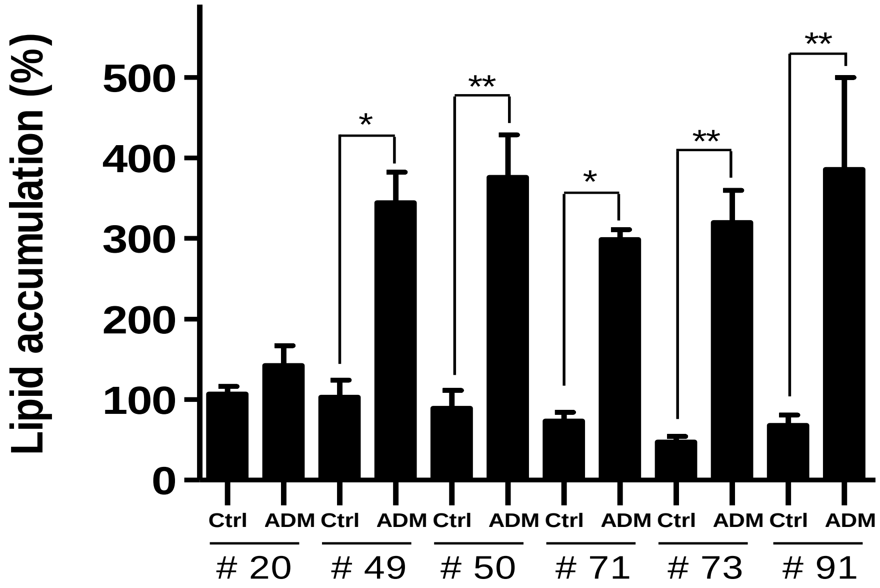

Figure S2. The adipogenic potential of ASCs from the donors (n=6). Post-confluent ASCs were cultured with or without of ADM for 14 days. The cells were then stained using Oil-red O (ORO). The relative intensity of lipid accumulation was quantified by extracting ORO stained lipid droplets with 100% isopropanol and optical density (OD) was measured at 495 nm. Values are expressed as mean ± SD of three independent experiments. $^{*}P < 0.05$ and $^{**}P < 0.01$ vs. undifferentiated parental ASCs. Ctrl: untreated group, ADM: adipogenic differentiation medium;

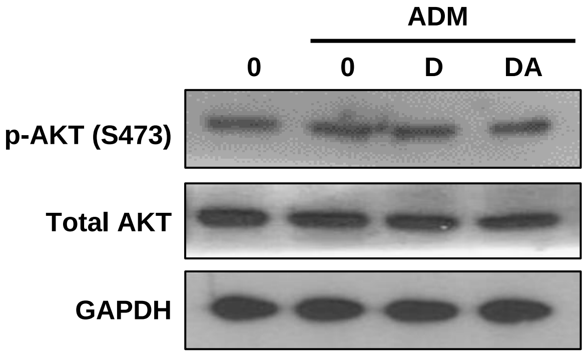

Figure S3. D and DA did not affect expression of phosphorylated AKT (S473). ASCs were cultured with ADM for 4 days in the presence or absence of D and DA.

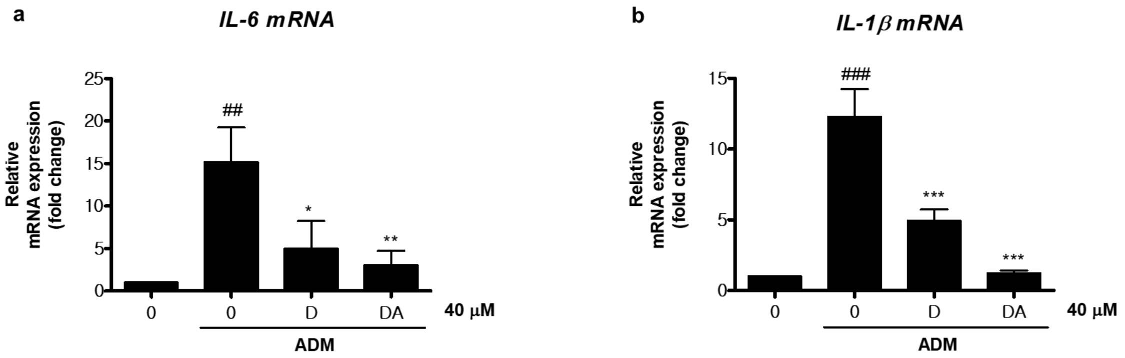

Figure S4. The effect of D and DA on the expression of inflammatory marker in ASCs. **(a,b)** The relative mRNA expression of inflammatory genes, *IL-6* and *IL-1β* analyzed by qRT-PCR on day 12 after ADM treatment. Values are expressed as mean ± SD of three independent experiments. $^{\#\#}P < 0.01$ and $^{\#\#\#}P < 0.001$ vs. undifferentiated group, $^{*}P < 0.05$, $^{**}P < 0.01$ and $^{***}P < 0.001$ vs. ADM treated group. ADM: adipogenic differentiation medium;
